# Predictive and prospective control strategies of elite batters during interception tasks: coupling of perception and action

**DOI:** 10.3389/fpsyg.2026.1775889

**Published:** 2026-03-05

**Authors:** Dukchan Jang

**Affiliations:** Department of Physical Education, Keimyung University, Daegu, Republic of Korea

**Keywords:** elite performance, interception, predictive control, prospective control, visuomotor coordination

## Abstract

**Introduction:**

Intercepting fast-moving objects, such as during baseball batting, requires the seamless integration of visual perception and motor execution under severe temporal constraints. Although the critical role of visual information in visuomotor coordination is well established, it remains unclear how predictive and prospective control interact when certain phases of visual input are unavailable. This study aimed to elucidate their distinct contributions by examining visuomotor coupling under selectively occluded early and/or late visual information during a time-constrained interception task.

**Methods:**

Fifteen skilled collegiate baseball players performed a computerized touchscreen interception task, intercepting a fast-moving stimulus (0.5, 0.67, and 1.0 m/s) at a target area. Stimulus visibility was manipulated across four occlusion conditions (full vision, early occlusion, late occlusion, and both occlusions). Eye and hand movements were recorded simultaneously to analyze gaze behavior, motor performance, and spatiotemporal coupling.

**Results:**

All temporal variables shortened as stimulus velocity increased. Under partial occlusion, participants employed distinct compensatory strategies: early occlusion accelerated movement initiation, whereas late occlusion adjusted completion timing. Hand timing error remained unaffected by occlusion, suggesting regulation by an internal timing model independent of immediate visual availability. In contrast, spatial accuracy (radial and spatial coupling errors) markedly declined when early predictive information was unavailable, particularly under both occlusions condition.

**Conclusion:**

These findings suggest that successful interception depends on dual control mechanisms—temporal efficiency maintained through flexible compensation and spatial accuracy governed by predictive planning based on early visual information. The concurrent loss of predictive and prospective control critically impairs spatial integration, underscoring the necessity of integrated feedforward-feedback coordination.

## 1 Introduction

Spectators of baseball are often impressed by elite batters’ remarkable ability to hit extremely fast-moving balls. Even when facing pitches that approach speeds testing the limits of human visuomotor control, expert batters execute highly precise eye movements (Bootsma and van Wieringen, 1990; Regan, 1997) and manage to make successful contact, even when the time available for visual information processing is severely constrained (Sarpeshkar and Mann, 2011; Sun and Jang, 2025). These interception behaviors raise fundamental questions about how sensory information is utilized to guide action under extreme temporal constraints. To explain such coordination, two major theoretical perspectives have been proposed: predictive control and prospective control. The predictive control perspective assumes that movement is largely pre-planned prior to execution, relying on early sensory cues to anticipate future states of the environment and to parameterize forthcoming actions (Davids et al., 2002; Dessing et al., 2005). In contrast, the prospective control perspective, grounded in the ecological approach, proposes that movement can be continuously regulated through online use of action-relevant sensory information available during execution itself (Beek et al., 2003; Montagne, 2005). Investigating how expert performers couple eye movements with motor execution during interception tasks may therefore offer critical insights into whether actions are guided primarily by early prediction, ongoing prospective regulation, or an integration of both from stimulus onset to the point of contact.

From the perspective of predictive control, interception tasks that unfold within extremely short timeframes require performers to pre-plan the initiation, duration, and amplitude of movement based on information perceived during the early phase of stimulus presentation (Dessing et al., 2005; Montagne, 2005). This view emphasizes the primacy of early perceptual judgments over continuous visual processing throughout the movement sequence (Bastin et al., 2006). Consistent with this account, research in high-speed ball sports has shown that it is virtually impossible for batters to maintain continuous gaze alignment with the ball throughout its flight (Kishita et al., 2020a; Tochikura et al., 2020). Instead, performers typically extract motion trajectory information early and execute predictive saccades toward anticipated points of contact (Croft et al., 2010; Kishita et al., 2020a; Mann et al., 2013). These findings suggest that, when intercepting fast-moving stimuli, sensory information can be processed in accordance with predictive control mechanisms. Within this framework, research on the quiet eye (QE) has emerged as a quantitative approach for investigating predictive visual strategies.

QE is defined as the final fixation or tracking gaze maintained on a specific location in the visuomotor workspace prior to the initiation of a motor response (Vickers, 2007, 2012; Vickers and Williams, 2007). In baseball-related tasks, QE is typically identified as the final gaze fixation on the target or its vicinity before movement onset. Early QE onset has been proposed as a marker of higher expertise (Vickers, 1996a), reflecting more efficient extraction of task-relevant information during a cognitive preparatory phase. Skilled performers are thought to fixate on critical information earlier, thereby allowing more time to parameterize subsequent motor commands such as movement direction, force, and velocity (McPherson and Vickers, 2004; Janelle et al., 2000; Vickers, 1996b). Empirical support for this view has been reported across sports. For example, Panchuk et al. (2016) showed that delayed QE onset in ice hockey goalkeepers was associated with poor performance during deflection saves. Collectively, these findings suggest that early visual information plays a crucial role in predictive movement initiation.

In contrast, the prospective perspective emphasizes the performer’s capacity to continuously utilize online sensory information, such as the evolving trajectory and velocity of a moving object, to regulate action throughout execution (Hoffman and Lenhard, 2004). From this viewpoint, neither the timing nor the spatial location of interception is fixed at movement onset. Instead, movement parameters are continuously adjusted based on perceptual input as the action unfolds, allowing for progressive refinement of accuracy (Dessing et al., 2005). This framework shifts the emphasis from advance prediction to the functional use of sensory information for online regulation.

Support for the prospective perspective has been demonstrated in interception tasks involving objects approaching at varying velocities. In such contexts, optical expansion—or tau—provides information about time-to-contact (Lee, 1974; Montagne et al., 1999; Savelsbergh et al., 1992). Studies of catching and interception have shown that performers adjust their movements in accordance with changes in tau, even during the late phases of action (Arzamarski et al., 2007). Electromyographic analyses further indicate that muscle activation patterns are modulated as a function of tau values during interception (Savelsbergh et al., 1992). These findings suggest that performers do not rely solely on pre-planned motor commands derived from early information, but instead continuously adapt their actions through prospective, online control.

Although predictive and prospective control have often been treated as discrete or competing mechanisms, growing evidence suggests that they operate as part of a continuum through context-dependent integration. Recent work has proposed that the visual system continuously generates predictions while simultaneously engaging in online adaptation to sensory input, with task-specific computations integrating internal state dynamics and environmental information (Márquez et al., 2024; Márquez and Treviño, 2025). From this hybrid perspective, visuomotor coordination is not the execution of a fixed motor plan, but an adaptive process in which feedforward and feedback processes jointly contribute to performance under uncertainty. Empirical studies support this view. For example, Land and McLeod (2000) showed that cricket batters employ predictive saccades to the anticipated bounce point while continuing to use online tracking information after the bounce. Similarly, Müller and Abernethy (2006) demonstrated that early visual information guides general movement planning, whereas information immediately before and after ball bounce is used to fine-tune temporal and spatial aspects of interception. These findings suggest that task-specific trajectory constraints—particularly the presence or absence of mid-trajectory discontinuities—may critically shape how predictive and prospective control processes are coordinated.

Despite this progress, much of the existing interception research has presented the full stimulus trajectory from initiation to completion, making it difficult to disentangle the relative contributions of early predictive information and late prospective information. Moreover, many interception paradigms involve mid-trajectory discontinuities (e.g., ball bounces), which may blur the functional boundary between early predictive planning and later prospective adjustment. In a recent study, Sun and Jang (2025) showed that experts maintain tighter eye-hand coupling than novices under late-occlusion conditions, indicating more efficient use of limited online information during the final execution phase. However, because early trajectory information was always available in that paradigm, it remained unclear whether superior interception performance reflected more efficient movement initiation strategies, more effective online correction, or an interaction of both. Consequently, the specific role of early visual information in establishing the cognitive preparatory phase—and its interaction with later online regulation—remains insufficiently understood.

To address this gap, it is essential to examine how restricting early visual information influences the extraction of motion dynamics and the subsequent fine-tuning of motor parameters. Limiting early trajectory information directly constrains the cognitive preparatory phase that underlies predictive movement initiation, thereby providing a critical test of predictive control mechanisms and their interaction with prospective regulation. By systematically manipulating visual access at different segments of the stimulus trajectory, it becomes possible to formalize how the availability of information defines an informational threshold at which control shifts between predictive planning and prospective adjustment.

Accordingly, the present study introduced four visual conditions: full vision, early occlusion, late occlusion, and both early and late occlusion. By employing a straight, non-bouncing trajectory under controlled laboratory conditions, the present paradigm minimizes trajectory discontinuities, thereby allowing clear attribution of observed behaviors to predictive and prospective control processes. From a predictive control perspective, eye and hand behavior should not differ substantially between the early-occluded and both-occluded conditions, as both equally restrict early predictive cues. From a prospective control perspective, similar behavioral patterns are expected between the late-occluded and both-occluded conditions, because late online information is absent in both. If predictive and prospective processes jointly contribute to interception, however, all four conditions should yield distinct patterns of gaze behavior and motor execution. In particular, the condition in which both early and late information are occluded is expected to produce the greatest impairment, reflecting the loss of both preparatory and online control processes.

This study aimed to clarify the contributions of predictive and prospective control by examining how the visual and motor systems are coupled when early and/or late visual information is restricted under time-constrained interception tasks.

## 2 Methods

### 2.1 Participants

Fifteen elite collegiate baseball players (age *M* = 21.35 years, *SD* = 1.91) from a university team in South Korea participated in this study. All participants were right-handed batters and throwers, as confirmed by self-report. Visual acuity was assessed using a standard Snellen chart, and all players had normal or corrected vision (20/20 or better). To ensure a high level of hitting performance, participants were required to have maintained a batting average of 0.280 or higher over the past two seasons in the KUSF University Baseball League. All players participated in team training sessions at least five times per week.

Participants were recruited through direct communication with the university team’s coaching staff, who facilitated voluntary participation. To confirm their eligibility, a formal screening process was conducted. Inclusion criteria were: (1) currently active membership of a collegiate baseball team, (2) no history of neurological or musculoskeletal disorders, (3) meeting the minimum batting average requirement; and (4) verified consistent attendance at team practices by the coaching staff.

An *a priori* power analysis (G*Power 3.1) was conducted to justify the sample size. Based on prior interception studies using comparable visuomotor paradigms (Sun and Jang, 2025; Sun et al., 2025), effect size was conservatively set to *f* = 0.30, reflecting medium effects typically observed for within-subject factors such as stimulus velocity and visual occlusion. With a significance level of 0.05, desired power of 0.95, and 12 repeated measurements (3 velocities × 4 occlusion conditions), the analysis indicated that a minimum of 13 participants was required. Accordingly, the sample size of 15 participants was deemed sufficient for the present study.

Before the experiment, all players were fully informed about the purpose and procedures of the study and provided written informed consent. The study was conducted in accordance with the ethical standards outlined in the Declaration of Helsinki and was approved by the Institutional Review Board of Keimyung University (IRB No. 40525-202409-HR-051-04).

### 2.2 Experimental task and apparatus

**Figure 1:**
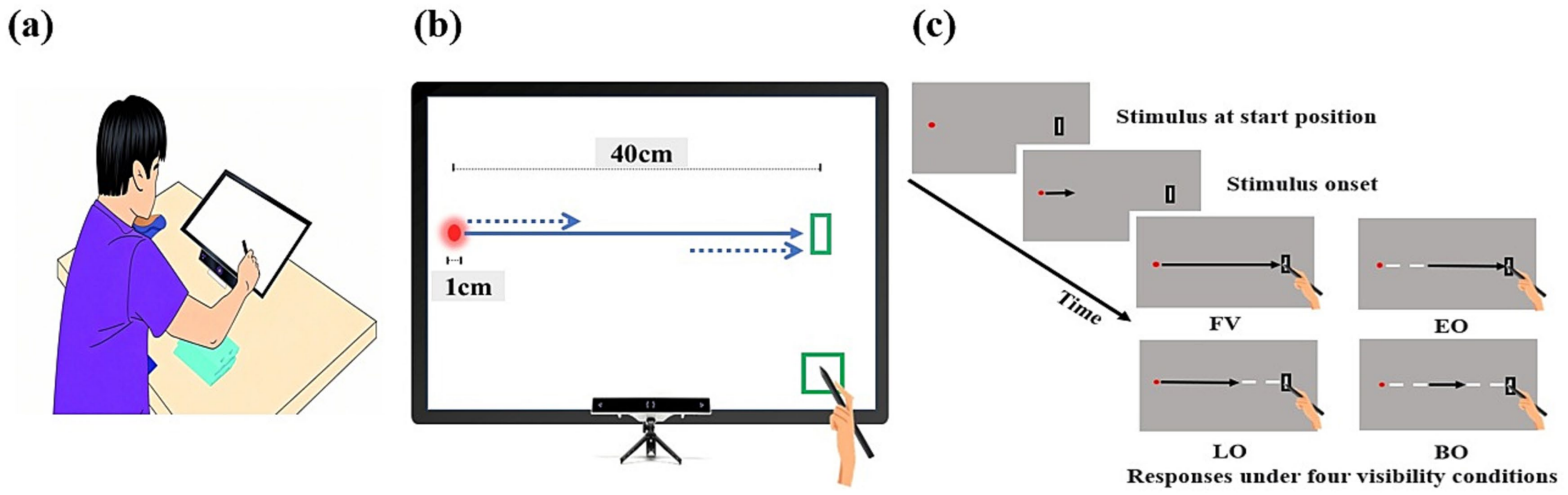
Experimental setup and task protocol. **(a)** Elite collegiate baseball players were seated in a stabilized posture using a chinrest and padded elbow supports to minimize extraneous movement and ensure a standardized reaching environment. The touchscreen was positioned at a fixed distance of 30 cm to maintain consistent reach kinematics and an optimal focal range of gaze tracking. **(b)** Stimuli were presented on the touchscreen, while a screen-mounted eye-tracker positioned below captured real-time gaze dynamics. The system synchronously recorded eye and hand (stylus) movements as participants intercepted the stimulus moving horizontally toward the top-right target area (3 cm × 9 cm). **(c)** Each trial was initiated by placing the stylus on the preparation area (bottom-right), triggering participants to direct their gaze to the starting point (left). After a 1.5 s delay, the stimulus traveled 40 cm at one of three velocities (0.5, 0.67, or 1.0 m/s), with visibility manipulated across four conditions: Full vision (FV), in which the entire trajectory remained visible; early occlusion (EO) and late occlusion (LO), in which the initial or final 13 cm (one-third of the path) was occluded, respectively; and both occlusions (BO), in which both the initial and final segments were occluded, leaving only the middle portion visible. Participants were instructed to intercept the stimulus with maximal spatial and temporal accuracy upon its entry into the target area.

The experimental setup was specifically designed to investigate the spatiotemporal coordination between eye and hand movements during a coincidence-anticipation interception task (see Figure 1). It comprised a gaze-tracking system, a touchscreen display, a chinrest apparatus, and a stylus. The touchscreen monitor had a diagonal screen size of 24 inches, a resolution of 1920 × 1080 pixels, and a refresh rate of 60 Hz. Participants were seated at a fixed viewing distance of 30 cm from the touchscreen display, with their heads stabilized by a chinrest. This distance allowed conversion of pixel coordinates to degree of visual angle (1° = 18.5 pixels) for consistent data analysis.

To record eye movements with high temporal and spatial resolution, the GP3 HD eye-tracking system (Gazepoint Inc., Vancouver, Canada) was utilized. This device was attached to the lower edge of the touchscreen monitor (U24OLED Edge HDMI, BitM Inc., Korea) and recorded binocular eye movements at a sampling rate of 150 Hz. A 150 Hz rate was adequate for accurately capturing the timing and large-scale dynamics (QE, saccade latency, smooth pursuit) required from time-to-contact analysis in this high-speed task. Gaze positions were calculated based on corneal reflections and pupil centers, with a 9-point calibration procedure performed at the start of each session. Both the eye-tracking system and the touchscreen were time-synchronized using a shared system clock, with a confirmed temporal accuracy of approximately ±6 ms.

Visual stimuli were presented on the touchscreen via a custom C# software program. Participants used a rubber-tipped stylus (14.5 cm length, 0.1 cm tip diameter, 22 g; MR-TP-01, Morac Co., Korea) held in their dominant hand to intercept a moving circular stimulus (1 cm diameter) by tapping the screen. Each trial began when the participant placed the stylus on a preparation area at the bottom-right corner of the screen and fixated on the stimulus’s starting position on the left edge. After a 1.5-s delay, the stimulus moved horizontally across a distance of 40 cm to a target area (3 cm × 9 cm) on the top-right of the screen. Participants were instructed to intercept the stimulus by tapping its center (or the closest center-line) as it entered the target area.

The experiment systematically varied two key factors: stimulus velocity and stimulus visibility. The stimulus was presented at one of three velocities—slow (0.5 m/s, ≈ 83 km/h real-world equivalent), medium (0.67 m/s, ≈ 111 km/h), and fast (1.0 m/s, ≈ 166 km/h) —and under one of four visual occlusion conditions: full vision, early occlusion, late occlusion, and both early and late occlusion. These four occlusion conditions were designed to systematically restrict visual information during the initial, final, or both the initial and final segments of the stimulus trajectory. All experimental conditions were randomized across trials. Participants were instructed to intercept the moving stimulus as accurately as possible. All eye and hand movement data, including temporal errors in milliseconds and spatial errors in *x*- and *y*-coordinates, were recorded automatically by the software from the start of the trial until the interception response was completed.

Eye movement data were processed offline to identify key oculomotor events. Saccades were detected using a velocity-based algorithm with a threshold of 30°/s and an acceleration threshold of 8,000°/s^2^ (Nyström and Holmqvist, 2010). Although higher sampling rates are generally required for micro-saccade analysis, the 150 Hz used here was sufficient to accurately identify the onset and offset of visually guided saccades (e.g., saccadic latency) relevant to the present task. To ensure the integrity of the analyzed data, a rigorous screening protocol was applied: trials were excluded if gaze-tracking loss exceeded 10% of the total trial duration (Holmqvist et al., 2011) or if technical artifacts (e.g., signal spikes or loss of stylus contact) were identified. Blinks were identified and removed, and any resulting minor data gaps were interpolated linearly to maintain temporal continuity without introducing artifacts. This data-cleaning process ensured that extracted spatiotemporal metrics reflected intentional visuomotor control rather than measurement noise.

### 2.3 Experimental procedure

Participants attended the laboratory individually for a single testing session. Upon arrival, they were given a brief rest period, after which the purpose and procedures of the study were thoroughly explained. Written informed consent was obtained prior to participation. To ensure participants fully understood the task, the researcher first demonstrated a sample trial. This was followed by 12 familiarization trials, during which participants used the stylus to intercept a moving stimulus on the touchscreen. These initial trials served to confirm participants’ comprehension of the task across conditions. Eye-tracker calibration was performed using a standard 9-point procedure in which participants fixated sequentially on nine cross-shaped targets displayed on the touchscreen. Calibration accuracy was rechecked every six trials, and recalibration was conducted as needed to maintain data reliability. Participants were seated in an adjustable chair with armrests, and their heads were stabilized using a chinrest to prevent head movement. With their elbows resting on padded supports, participants maintained a stable and comfortable posture throughout the task. Before the main experimental session, participants completed 24 practice trials—2 for each of the 12 combinations of velocity and occlusion conditions (2 × 12 = 24 trials)—to ensure full adaptation to the task and apparatus.

Each practice trial began with a verbal “ready” cue from the experimenter. Participants placed the stylus on the designated preparation area located at the bottom-right corner of the touchscreen and directed their gaze to the stimulus’s starting position at the left edge of the screen (see Figure 1). Once contact was made with the preparation area, a circular stimulus (1 cm diameter) appeared at the starting point. After a 1.5-s delay, the stimulus moved horizontally toward the target area (3 cm × 9 cm) located at the top-right portion of the screen. The stimulus traveled a distance of 40 cm under one of three velocity conditions—slow (0.5 m/s), medium (0.67 m/s), or fast (1.0 m/s)—and one of four occlusion conditions. Participants were instructed to intercept the stimulus with the stylus as accurately as possible in both space and time as it entered the target area. Upon stylus-screen contact, the moving stimulus immediately disappeared, marking the end of the trial. The four occlusion conditions were as follows: In the full vision condition, the entire 40 cm trajectory of the stimulus was visible. In the early occlusion condition, the first 13 cm of the trajectory (approximately one-third) was occluded, corresponding to a temporal loss of the initial 260 ms (at 0.5 m/s), 194 ms (at 0.67 m/s), or 130 ms (at 1.0 m/s). In the late occlusion condition, the final 13 cm was occluded. Lastly, in both occlusions condition, both the initial and final 13 cm segments of the trajectory were occluded, leaving only the middle portion visible. Although the stimulus always traveled from left to right toward the target area, participants were informed in advance that its movement direction could vary slightly to prevent over-reliance on a fully predictable trajectory.

Following the practice phase, participants completed 120 experimental trials, comprising 10 trials for each of the 12 combinations of velocity and occlusion conditions (10 × 12 = 120 trials). The trial order was randomized using a custom-generated Excel randomization sequence, and participants were unaware of the trial order in advance. The procedure for the experimental trials was identical to that of the practice trials, including stimulus presentation and interception response. To avoid influencing participants’ natural control strategies, no performance feedback was provided during the experiment. A brief one-minute rest period was given after every 12 trials, during which participants remained seated in the chinrest with their elbows supported. Upon completing all trials, participants received a short debriefing regarding the purpose of the study.

### 2.4 Data analysis

The collected data were used to compute several variables to investigate visuomotor coordination strategies during the interception task. These variables were categorized into three domains: (1) eye movement characteristics, (2) spatiotemporal accuracy and response timing of hand movements, and (3) eye-hand coupling.

#### 2.4.1 Eye movement characteristics

(a) QE (ms): Based on Vickers (2007), QE was operationally defined as a period of stable tracking gaze on the stimulus’s starting point or its vicinity. The onset of QE was defined as the moment when tracking gaze began within 3° of visual angle for at least 100 ms (15 frames) before the hand movement initiated toward the target area. The offset was defined as the moment the tracking gaze moved outside of this 3° visual angle for at least 100 ms. Importantly, QE onset could occur prior to stimulus motion onset, as gaze was often stabilized at the stimulus’s starting location before the appearance of the moving stimulus. Therefore, QE was the time interval between QE onset and QE offset, and QE duration may include time both before and after stimulus onset, reflecting the duration of the cognitive preparatory phase crucial for extracting motion trajectory information and fine-tuning subsequent motor parameters before motor initiation. (b) Saccadic latency (ms): This was the time from stimulus onset (i.e., the initial appearance of the moving stimulus at its starting position) to the initiation of the first saccadic eye movement. Because QE could begin prior to stimulus onset, saccadic latency was temporally distinct from QE and did not include the QE period. This period reflects the perceptual encoding and initial decision-making time used to plan the initial eye and hand movements, serving as a key indicator of the efficiency and speed of the early visuomotor system (Hayhoe et al., 2012; Kishita et al., 2020b). (c) Gaze duration at the target area (ms): This was defined as the time interval between the moment the gaze arrived at the target area and the moment the stimulus entered that area. It provides information on the temporal relationship between eye movements and stimulus characteristics at the final interception phase. (d) Gaze error at the target area (mm): This variable was used to quantify the spatial relationship between eye movements and stimulus characteristics during the final stage of interception. It measured the spatial discrepancy (i.e., radial error) between the final gaze position and the stimulus at the target area at the moment of response completion. This was calculated as the Euclidean distance using the following formula:

*Gaze error* = $\;\sqrt{{\left(x_{\text{final gaze}}-x_{\text{stimulus endpoint}}\right)}^{2}+{\left(y_{\text{final gaze}}-y_{\text{stimulus endpoint}}\right)}^{2}}$

where *x* and *y* denote the horizontal and vertical deviations, respectively, between the final gaze location and the stimulus endpoint.

#### 2.4.2 Spatiotemporal error and response timing

(a) Timing error at the target area (ms): This was defined as the absolute difference in time between the moment the stimulus reached the target area and the moment the stylus made contact with the touchscreen, reflecting the temporal accuracy of the interception. (b) Radial error at the target area (mm): This was defined as the spatial discrepancy (i.e., Euclidean distance) between the location of the stimulus and the point of stylus contact at the target area. This variable quantifies the spatial accuracy of the interception. The error was calculated using the following formula:

*Radial error* = $\;\sqrt{{\left(x_{\text{stimulus endpoint}}-x_{\text{stylus contact}}\right)}^{2}+{\left(y_{\text{stimulus endpoint}}-y_{\text{stylus contact}}\right)}^{2}}$

where *x* and *y* denote the horizontal and vertical deviations, respectively, between the stimulus endpoint and the stylus contact point. (c) Reaction time (ms): This was the time elapsed from stimulus onset at the starting point to the moment the stylus was lifted from the preparation area, reflecting the perceptual and cognitive decision-making time needed to initiate the motor response. (d) Movement time (ms): This was the time interval from the initiation of hand movement (i.e., lifting the stylus) to the completion of the response at the target area, reflecting the speed and efficiency of motor execution, which integrates both pre-planned control and online adjustments.

#### 2.4.3 Eye-hand coupling

(a) Timing coupling (ms): This variable was computed by subtracting the gaze arrival time at the target area from the time of stylus contact. It quantifies the time lag between the visual and motor systems at the point of interception. (b) Spatial coupling (mm): This variable captured the degree of spatial alignment between the gaze and the hand at the completion of the response. It was calculated as the Euclidean distance between the final gaze position and the stylus contact point using the following formula:

*Spatial coupling* = $\sqrt{{\left(x_{\text{final gaze}}-x_{\text{stylus endpoint}}\right)}^{2}+{\left(y_{\text{final gaze}}-y_{\text{stylus endpoint}}\right)}^{2}}$

where *x* and *y* denote the horizontal and vertical deviations, respectively, between the gaze location and the stylus endpoint.

A two-way repeated measures analysis of variance (ANOVA) was conducted to examine the effects of stimulus velocity and stimulus visibility (operationalized as occlusion conditions) on each dependent variable. The within-subjects factors were velocity (0.5, 0.67, and 1.0 m/s) and occlusion condition (full vision, early occlusion, late occlusion, and both occlusions). When the assumption of sphericity was violated, the Greenhouse–Geisser correction was applied. Bonferroni-adjusted post-hoc comparisons were conducted where appropriate to control for Type I error. The level of statistical significance was set at *α* = 0.05 for all tests. Analyses were performed using IBM SPSS Statistics (Version 30). Results are reported as means ± standard errors, and the effect size for all ANOVA results is reported using partial eta-squared (*η*_p_^2^).

## 3 Results

### 3.1 Eye movement characteristics

#### 3.1.1 QE

Significant main effects were found for both stimulus velocity, *F*(2,28) = 64.84, *p* < 0.001, *η*_p_^2^ = 0.82, and occlusion condition, *F*(3,42) = 8.97, *p* < 0.001, *η*_p_^2^ = 0.39. Post-hoc tests for the main effect of stimulus velocity revealed that QE significantly decreased as stimulus velocity increased (*p* < 0.001). For the main effect of occlusion, post-hoc comparisons indicated that the full vision condition had a significantly longer QE than the other conditions (*p* < 0.05), and the late occlusion condition resulted in a longer QE than the early occlusion condition (*p* < 0.05).

**Figure 2:**
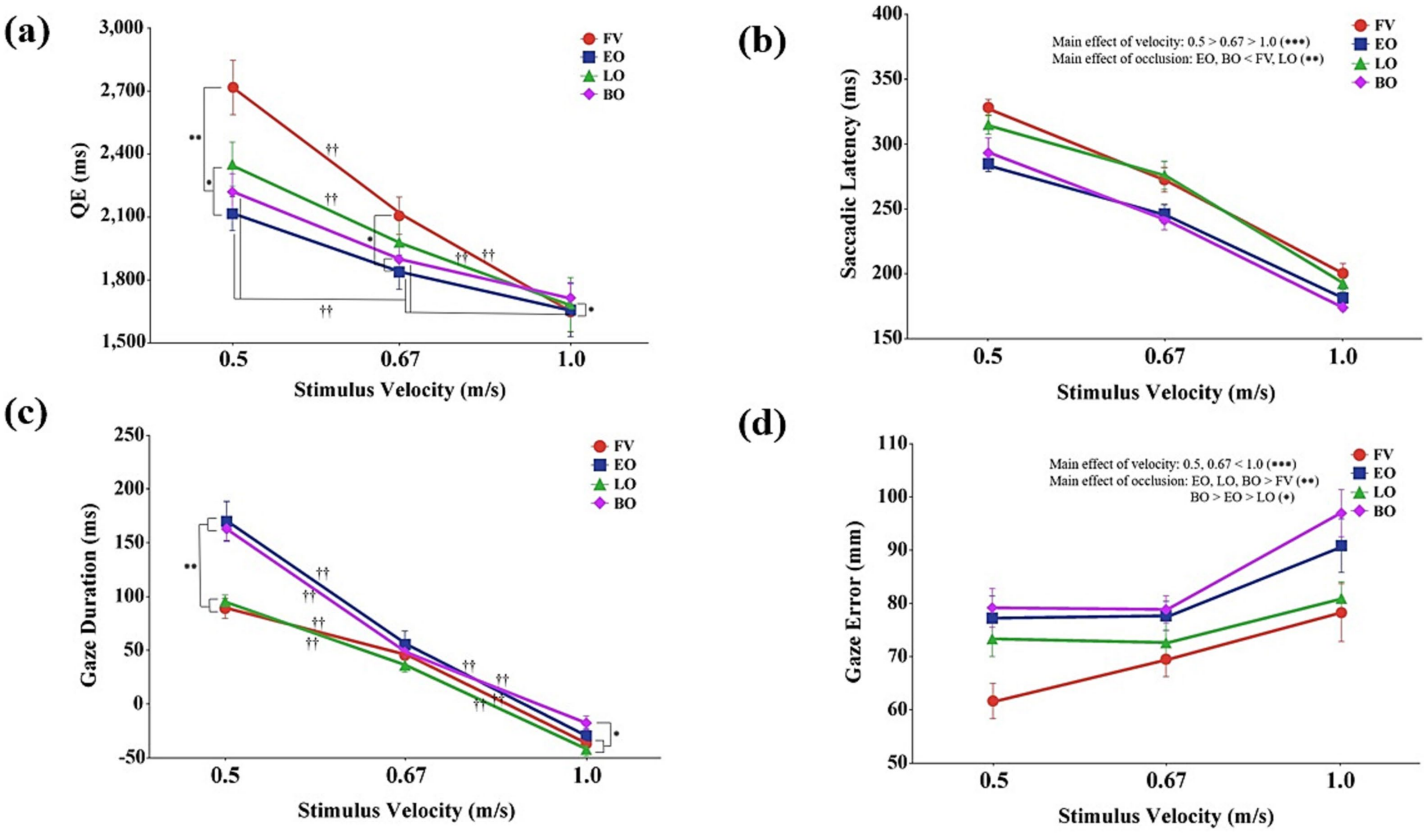
Eye-movement characteristics as a function of stimulus velocity and occlusion condition. **(a)** Quie eye (QE) (ms); **(b)** Saccadic latency (ms); **(c)** Gaze duration (ms); **(d)** Gaze error (mm). Data are presented as a function of stimulus velocity (0.5, 0.67, and 1.0 m/s) and occlusion condition (FV, full vision; EO, Early occlusion; LO, late occlusion; BO, both occlusions), where statistically significant effects were observed. Error bars represent standard errors of the mean (SEM). Asterisks and daggers indicate significant differences (^*^p < 0.05, ^**^p < 0.01, ^††^*p* < 0.01).

A significant interaction was also found between stimulus velocity and occlusion condition, *F*(6,84) = 3.93, *p* < 0.01, *η*_p_^2^ = 0.22 (Figure 2a). Post-hoc comparisons examining the simple main effects of occlusion condition at each velocity showed that at slow velocity (0.5 m/s), the full vision condition resulted in a significantly longer QE than all other conditions (*p* < 0.01). Furthermore, the late occlusion condition had a longer QE than the early occlusion condition (*p* < 0.05). At medium velocity (0.67 m/s), the full vision condition had a longer QE than early and both occlusions conditions (*p* < 0.05). At fast velocity (1.0 m/s), the full vision condition showed a longer QE than the early occlusion condition (*p* < 0.05). Additionally, post-hoc comparisons examining the simple main effects of stimulus velocity within each occlusion condition revealed that for the full vision and late occlusion conditions, QE decreased as stimulus velocity increased (*p* < 0.01). For early and both occlusions conditions, QE decreased at both 0.67 m/s and 1.0 m/s compared to 0.5 m/s (*p* < 0.01).

#### 3.1.2 Saccadic latency

Significant main effects were found for both stimulus velocity, *F*(2,28) = 127.69, *p* < 0.001, *η*_p_^2^ = 0.90, and occlusion condition, *F*(3,42) = 14.25, *p* < 0.001, *η*_p_^2^ = 0.50 (Figure 2b). Post-hoc tests for the main effect of stimulus velocity revealed that saccadic latency significantly decreased as stimulus velocity increased (*p* < 0.001). For the main effect of occlusion, post-hoc comparisons indicated that early and both occlusions conditions had significantly shorter saccadic latencies than the full vision and late occlusion conditions (*p* < 0.01). No significant interaction effect, *F*(6,84) = 0.84, *p* = 0.54, *η*_p_^2^ = 0.06 was observed.

#### 3.1.3 Gaze duration

Significant main effects were found for both stimulus velocity, *F*(2,28) = 388.28, *p* < 0.001, *η*_p_^2^ = 0.96, and occlusion condition, *F*(3,42) = 10.05, *p* < 0.01, *η*_p_^2^ = 0.42. Post-hoc tests for the main effect of stimulus velocity revealed that gaze duration significantly decreased as stimulus velocity increased (*p* < 0.001). For the main effect of occlusion, post-hoc comparisons indicated that the full vision and late occlusion conditions showed significantly shorter gaze durations than early and both occlusions conditions (*p* < 0.01).

A significant interaction between stimulus velocity and occlusion condition was also observed, *F*(6,84) = 5.93, *p* < 0.001, *η*_p_^2^ = 0.30 (Figure 2c). Post-hoc comparisons examining the simple main effects of occlusion condition at each velocity revealed that at slow velocity (0.5 m/s), the full vision and late occlusion conditions resulted in significantly shorter gaze durations than early and both occlusions conditions (*p* < 0.01). At medium velocity (0.67 m/s), no significant differences were found among the occlusion conditions (*p* > 0.05). At fast velocity (1.0 m/s), the full vision and late occlusion conditions showed shorter gaze durations than both occlusions condition (*p* < 0.05). Additional post-hoc analyses examining the simple main effects of stimulus velocity within each occlusion condition showed that in all occlusion conditions, gaze duration significantly decreased as stimulus velocity increased (*p* < 0.01).

#### 3.1.4 Gaze error

Significant main effects were found for both stimulus velocity, *F*(2,28) = 50.56, *p* < 0.001, *η*_p_^2^ = 0.78, and occlusion condition, *F*(3,42) = 25.20, *p* < 0.001, *η*_p_^2^ = 0.64 (Figure 2d). Post-hoc tests for the main effect of stimulus velocity revealed that gaze error at the target area was significantly greater in the fast velocity (1.0 m/s) condition compared to both the slow (0.5 m/s) and medium (0.67 m/s) conditions (*p* < 0.001). For the main effect of occlusion, gaze error was significantly smaller in the full vision condition than in all occlusion conditions (*p* < 0.01). Moreover, gaze error systematically varied across occlusion conditions, with the largest errors observed under both occlusions condition, followed by early occlusion, and then late occlusion (*p* < 0.05). However, no significant interaction effect, *F*(6,84) = 1.23, *p* = 0.30, *η*_p_^2^ = 0.08 was observed.

### 3.2 Spatiotemporal error and response timing

#### 3.2.1 Timing error

**Figure 3:**
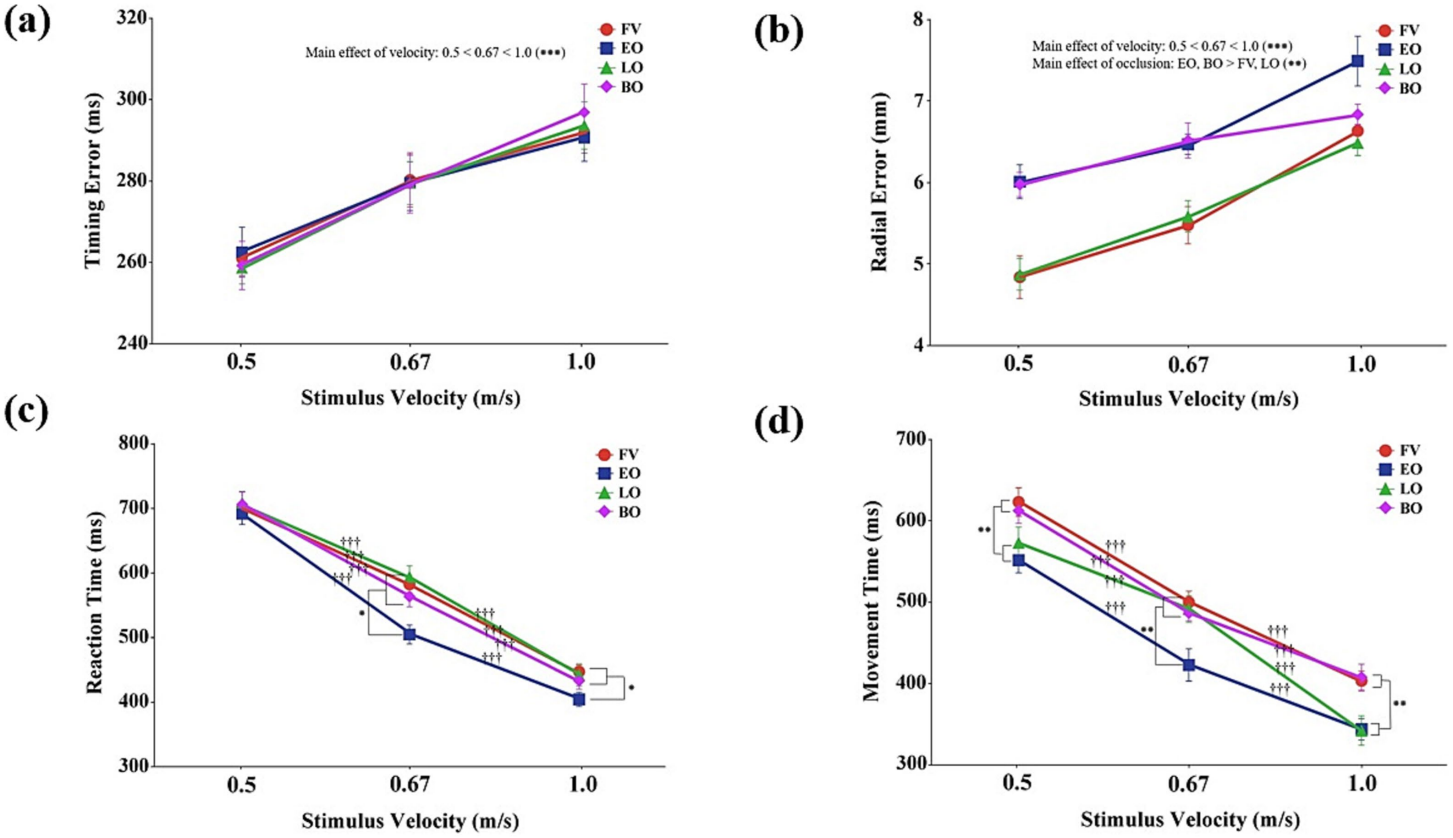
Spatiotemporal accuracy and response timing as a function of stimulus velocity and occlusion condition. **(a)** Timing error (ms); **(b)** Radial error (mm); **(b)** Reaction time (ms); **(d)** Movement time (ms). Data are presented as a function of stimulus velocity (0.5, 0.67, and 1.0 m/s) and occlusion condition (FV, full vision; EO, early occlusion; LO, late occlusion; BO, both occlusions), where statistically significant effects were observed. Error bars represent standard errors of the mean (SEM). Asterisks and daggers indicate significant differences (^*^p < 0.05, ^**^p < 0.01, ^†††^*p* < 0.001).

A significant main effect of stimulus velocity was found for hand movement timing error, *F*(2,28) = 53.33, *p* < 0.001, *η*_p_^2^ = 0.79 (Figure 3a). Post-hoc tests for the main effect of stimulus velocity revealed that timing error significantly increased with faster stimulus velocities (*p* < 0.001). Neither the main effect of occlusion condition, *F*(3,42) = 0.38, *p* = 0.99, *η*_p_^2^ = 0.01, nor the interaction between stimulus velocity and occlusion condition, *F*(6,84) = 0.41, *p* = 0.87, *η*_p_^2^ = 0.03, was statistically significant.

#### 3.2.2 Radial error

A significant main effect was found for both stimulus velocity, *F*(2,28) = 168.83, *p* < 0.001, *η*_p_^2^ = 0.92, and occlusion condition, *F*(3,42) = 21.87, *p* < 0.001, *η*_p_^2^ = 0.61 (Figure 3b). Post-hoc tests for the main effect of stimulus velocity revealed that the spatial error of hand movements significantly increased as stimulus velocity increased (*p* < 0.001). For the main effect of occlusion, post-hoc comparisons indicated that the full vision and late occlusion conditions resulted in significantly smaller radial errors than early and both occlusions conditions (*p* < 0.01). However, the interaction between stimulus velocity and occlusion condition, *F*(6,84) = 1.41, *p* = 0.22, *η*_p_^2^ = 0.09 was not significant.

#### 3.2.3 Reaction time

Significant main effects were found for both stimulus velocity, *F*(2,28) = 523.99, *p* < 0.001, *η*_p_^2^ = 0.97, and occlusion condition, *F*(3,42) = 11.92, *p* < 0.001, *η*_p_^2^ = 0.46. Post-hoc tests for the main effect of stimulus velocity revealed that reaction time significantly decreased as stimulus velocity increased (*p* < 0.001). For the main effect of occlusion, post-hoc comparisons indicated that reaction time was significantly shorter in the early occlusion condition compared to the other occlusion conditions (*p* < 0.01).

A significant interaction was also found between stimulus velocity and occlusion condition, *F*(6,84) = 3.10, *p* < 0.01, *η*_p_^2^ = 0.18 (Figure 3c). Post-hoc comparisons examining the simple main effects of occlusion condition at each velocity showed that at slow velocity (0.5 m/s), no significant differences were observed among the occlusion conditions (*p* > 0.05). At both medium (0.67 m/s) and fast velocities (1.0 m/s), however, reaction time was significantly shorter in the early occlusion condition compared to the other conditions (*p* < 0.05). Additionally, post-hoc analyses of the simple main effects of stimulus velocity within each occlusion condition revealed that reaction time significantly decreased as stimulus velocity increased across all conditions (*p* < 0.001).

#### 3.2.4 Movement time

Significant main effects were found for both stimulus velocity, *F*(2,28) = 258.49, *p* < 0.001, *η*_p_^2^ = 0.95, and occlusion condition, *F*(3,42) = 10.50, *p* < 0.001, *η*_p_^2^ = 0.43. Post-hoc tests for the main effect of stimulus velocity revealed that movement time significantly decreased as stimulus velocity increased (*p* < 0.001). For the main effect of occlusion, post-hoc comparisons indicated that movement time was significantly shorter in the early and late occlusion conditions compared to full vision and both occlusions conditions (*p* < 0.05), with the shortest movement time observed in the early occlusion condition.

A significant interaction was also found between stimulus velocity and occlusion condition, *F*(6,84) = 3.12, *p* < 0.01, *η*_p_^2^ = 0.18 (Figure 3d). Post-hoc comparisons examining the simple main effects of occlusion condition at each velocity showed that at both slow (0.5 m/s) and fast velocities (1.0 m/s), movement time was significantly shorter in the early and late occlusion conditions compared to full vision and both occlusions conditions (*p* < 0.01). At medium velocity (0.67 m/s), movement time was significantly shorter in the early occlusion condition compared to the other conditions (*p* < 0.01). Furthermore, across all occlusion conditions, movement time consistently decreased as stimulus velocity increased (*p* < 0.001).

### 3.3 Eye-hand coupling

#### 3.3.1 Temporal coupling

A significant main effect of stimulus velocity was found for temporal coupling, *F*(2,28) = 123.16, *p* < 0.001, *η*_p_^2^ = 0.90. Post-hoc tests revealed that temporal coupling significantly decreased as stimulus velocity increased (*p* < 0.001). The main effect of occlusion condition was not statistically significant, *F*(3,42) = 1.57, *p* = 0.21, *η_p_*^2^ = 0.10.

**Figure 4:**
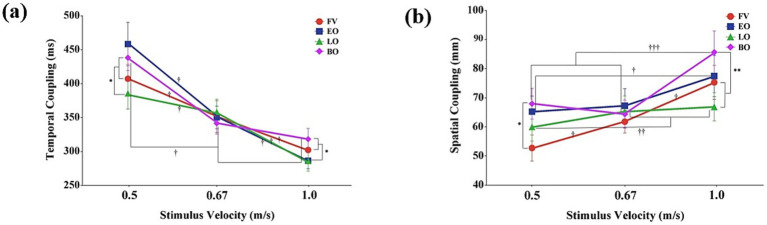
Eye-hand coupling as a function of stimulus velocity and occlusion condition. **(a)** Temporal coupling (ms); **(b)** Spatial coupling (mm). Data are presented as a function of stimulus velocity (0.5, 0.67, and 1.0 m/s) and occlusion condition (FV, full vision; EO, early occlusion; LO, late occlusion; BO, both occlusions), where statistically significant effects were observed. Error bars represent standard errors of the mean (SEM). Asterisks and daggers indicate significant differences (^*^p < 0.05, ^**^p < 0.01, ^†^*p* < 0.05, ^††^*p* < 0.01, ^†††^*p* < 0.001).

However, a significant interaction was observed between stimulus velocity and occlusion condition, *F*(6,84) = 2.88, *p* < 0.05, *η*_p_^2^ = 0.17 (Figure 4a). Post-hoc comparisons examining the simple main effects of occlusion condition at each velocity showed that at slow (0.5 m/s) and fast velocities (1.0 m/s), temporal coupling was significantly longer in full vision and both occlusions conditions compared to the late occlusion condition (*p* < 0.05). Conversely, at the medium velocity (0.67 m/s), no significant differences were found among the occlusion conditions (*p* > 0.05). Additionally, post-hoc analyses examining the simple main effects of stimulus velocity within each occlusion condition showed that temporal coupling significantly decreased as stimulus velocity increased across the full vision, early occlusion, and late occlusion conditions (*p* < 0.05). In both occlusions condition, temporal coupling was significantly shorter at both 0.67 m/s and 1.0 m/s compared to 0.5 m/s (*p* < 0.05).

#### 3.3.2 Spatial coupling

Significant main effects were found for both stimulus velocity, *F*(2,28) = 49.08, *p* < 0.001, *η*_p_^2^ = 0.78, and occlusion condition, *F*(3,42) = 9.73, *p* < 0.001, *η*_p_^2^ = 0.41. Post-hoc tests for the main effect of stimulus velocity revealed that spatial coupling significantly increased as stimulus velocity increased (*p* < 0.01). For the main effect of occlusion, post-hoc comparisons indicated that spatial coupling was significantly larger in early occlusion and both occlusions conditions compared to the full vision and late occlusion conditions (*p* < 0.01).

A significant interaction was also found between stimulus velocity and occlusion condition, *F*(6,84) = 3.39, *p* < 0.01, *η_p_*^2^ = 0.19 (Figure 4b). Post-hoc comparisons examining the simple main effects of occlusion condition at each velocity showed that at slow velocity (0.5 m/s), spatial coupling was smallest in the full vision condition, followed by the early and late occlusion conditions, and was largest in both occlusions condition (*p* < 0.05). At medium velocity (0.67 m/s), no significant differences were found among the occlusion conditions (*p* > 0.05). At fast velocity (1.0 m/s), spatial coupling was significantly smaller in the full vision and late occlusion conditions compared to both occlusions condition (*p* < 0.01). Additionally, post-hoc analyses examining the simple main effects of stimulus velocity within each occlusion condition showed that spatial coupling significantly increased as stimulus velocity increased for all conditions. Specifically, spatial coupling increased progressively across all velocity levels in the full vision condition (*p* < 0.05). For the early occlusion condition, spatial coupling at 1.0 m/s was significantly greater than at 0.5 m/s (*p* < 0.05). For the late occlusion condition, spatial coupling at both 0.67 m/s and 1.0 m/s was significantly greater than at 0.5 m/s (*p* < 0.01). Finally, for both occlusions condition, spatial coupling at 1.0 m/s was significantly greater than at both 0.5 m/s and 0.67 m/s (*p* < 0.001).

## 4 Discussion

Intercepting a moving object requires precise and accurate responses to the object’s arrival time and location, necessitating sophisticated visuomotor coordination (Mann et al., 2013; Vickers, 2007). This study analyzed the coupling process between the visual and motor systems under conditions of temporal pressure and informational uncertainty, which were manipulated by blocking early and late stimulus information. This study aimed to clarify the roles of pre-planning (i.e., predictive control strategy) and online control (i.e., prospective control strategy) in visuomotor information processing. In a controlled laboratory task mimicking a baseball hitting scenario, participants used a stylus to accurately intercept a fast-moving stimulus as it reached a designated target area. The experiment manipulated stimulus visibility across four conditions—full vision, early occlusion, late occlusion, and both occlusions—to systematically vary uncertainty. We also modulated stimulus velocity at three levels (slow: 0.5 m/s, medium: 0.67 m/s, and fast: 1.0 m/s) to add temporal constraints on both reaction and movement times. Our results revealed a complex and synergistic interplay between stimulus velocity and visual information occlusion that impacted visuomotor coordination. This interplay aligns with established visuomotor frameworks suggesting that the onset and magnitude of optimal interception strategies are highly dependent on task parameters such as target speed and the predictability of the trajectory (Márquez and Treviño, 2024a). Because participants consisted of highly trained baseball players, the observed patterns reflect control strategies within an expert sample rather than serving as direct evidence of expertise-specific effects relative to novices. Crucially, our results demonstrated that participants employed strategic, compensatory adjustments—such as accelerating initiation under the early occlusion condition or prioritizing completion time under the late occlusion condition—to maintain temporal efficiency under visual uncertainty. However, the effectiveness of these strategies was limited; when both predictive and prospective control were simultaneously constrained in both occlusions condition, a critical breakdown in spatiotemporal accuracy emerged. These findings further support the view that successful visuomotor interception depends on the integrated operation of predictive and prospective control mechanisms to achieve effective spatiotemporal coordination.

All temporal variables analyzed in this study—including QE, saccadic latency, gaze duration, reaction time, movement time, and temporal coupling—exhibited a consistent trend of shortening or decreasing as stimulus velocity increased (Figures 2, 3, 4a). This demonstrates that temporal pressure served as a major constraint, effectively compressing the entire visuomotor system’s phases, encompassing cognitive preparation (Land and Furneaux, 1997; Mann et al., 2013; Vickers, 2007), motor initiation (Fooken and Spering, 2019), and the final synchronization interval (Tresilian, 1999). Such temporal compression reflects the brain’s ability to entrain visuomotor responses to speed fluctuations, where gaze and manual metrics synchronize with the dynamics of the target to minimize sensory prediction errors (Treviño and Márquez, 2025a,b). Most notably, across the medium velocity (0.67 m/s, ≈ 111 km/h) condition, there were no significant differences among the occlusion conditions for measures of gaze duration, temporal coupling, and spatial coupling (Figures 2c, 4a,b). This indicates that within this expert sample, participants were able to apply well-established internal models and automatized strategies at this velocity, resulting in stable performance even under varying uncertainty (Land and McLeod, 2000). Conversely, at the relatively less familiar slow and fast velocities, participants displayed distinct strategic adaptation patterns in response to changes in visual information constraints.

The visual system demonstrated a clear strategic trade-off between predictive planning and prospective adjustment in response to information uncertainty (Sun and Jang, 2025). The significant shortening of QE and saccadic latency observed in early occlusion and both occlusions conditions (Figures 2a,b) suggests that the absence of initial trajectory information effectively curtailed the predictive planning stage. Consequently, participants adopted a compensatory adaptation by rapidly shifting gaze to the target area, attempting to prioritize online control through fovea-based information processing (de Brouwer and Spering, 2022). These anticipatory gaze shifts toward future target positions reflect the visual system’s reliance on statistical regularities to guide behavior when continuous tracking is disrupted (Treviño and Márquez, 2025b). Concurrently, the gaze duration was significantly prolonged in the early occlusion condition (Figure 2c), indicating the participants’ strategic effort to maximize the duration of online visual feedback to offset the loss of early planning time. This adaptive modulation of gaze under uncertainty might also be reflected in physiological markers such as pupillary responses, which have been shown to predict the effectiveness of interception strategies (Márquez and Treviño, 2024b). However, even with this highly trained group, these compensatory adjustments were insufficient, as gaze error increased across all occlusion conditions, peaking most notably in the both occlusions condition (Figure 2d). This outcome implies that the loss of predictive control cannot be fully compensated by the mere prolongation of gaze fixation, underscoring the critical role of early visual information acquisition in maintaining overall performance accuracy (Aoyama et al., 2022). While the current study focused on condition-based differences in gaze and interception performance, future research employing correlational or regression analyses could further clarify the direct functional link between gaze error and specific spatiotemporal interception errors, particularly under conditions of high informational uncertainty.

The hand movement analysis clearly demonstrated that temporal control and spatial control are governed by distinct visuomotor mechanisms. Reaction time was significantly shortened in the early occlusion condition, while movement time was reduced in both the early occlusion and late occlusion conditions (Figures 3c,d), indicating a strategic acceleration across both initiation and execution phases of hand movement to compensate for informational constraints (Márquez et al., 2024). Nevertheless, the finding that timing error was unaffected by occlusion (Figure 3a) and influenced only by stimulus velocity suggests that temporal control relied on stable feedforward predictions. This pattern reflects a robust internal timing model that operates largely independent of moment-to-moment visual information (Franklin and Wolpert, 2011), rather than indicating an expertise-exclusive mechanism per se. The selective degradation of spatial accuracy despite this preserved temporal precision points toward a dissociation between internal models of timing and spatial integration, potentially highlighting the role of cerebellar feedforward control in maintaining temporal consistency under uncertainty (Miall and Wolpert, 1996; Tanaka et al., 2020). In contrast, the radial error, which reflects spatial accuracy, was significantly impaired in conditions lacking initial information (i.e., early and both occlusions) (Figure 3b). This finding indicates that even highly practiced performers depend critically on early spatial information to maintain spatial precision, highlighting that, in temporally constrained tasks, the hand’s spatial control appears to rely on the availability of early predictive information (Land and McLeod, 2000).

The analysis of eye-hand temporal coupling demonstrated that the visuomotor system can achieve equivalent temporal synchronization efficiency through distinct strategic pathways (Figure 4a). Both the early occlusion and late occlusion conditions successfully compressed the coupling interval to a similar extent. Notably, at the 0.5 m/s condition, the absence of a significant difference between early and late occlusion in temporal coupling may reflect the reciprocal nature of their compensatory adjustments. In the early occlusion condition, an early saccadic onset was balanced by accelerated hand movement initiation and execution, whereas the late occlusion condition appeared to compensate for delayed saccadic shifts through a prioritized completion strategy. These divergent yet effective adaptations resulted in a comparable level of temporal synchronization efficiency across both occlusion types. Specifically, the early occlusion condition utilized an accelerated initiation strategy, whereas the late occlusion condition relied on an adjusted completion strategy. Despite these distinct approaches, both maintained efficient temporal integration under informational constraints. In contrast, as observed at slow and fast velocities, the prolonged temporal coupling in the full vision and both occlusions conditions stemmed from different sources. In the full vision condition, the extended coupling may reflect a relatively more conservative control strategy, possibly aimed at maintaining a temporal buffer through continuous visual feedback. Conversely, the prolonged coupling in the both occlusions condition appears to result from a breakdown in the synchronization process caused by the simultaneous loss of both predictive and prospective control mechanisms (Franklin and Wolpert, 2011), leading to unstable and inefficient temporal integration. This breakdown highlights a limit in compensatory capacity within the tested visuomotor system, rather than a failure unique to or diagnostic of expert performance, emphasizing the boundary conditions under which adaptive control can be sustained.

The strategic success observed in temporal coupling clearly revealed its limitations in spatial integration. The spatial coupling error was highest in the both occlusions condition (Figure 4b), suggesting that the dual loss of predictive planning and online adjustment disrupted the system’s spatial integration (Márquez et al., 2024; Mrotek and Soechting, 2007; Sun and Jang, 2025). Despite the temporal success observed in the early occlusion condition (i.e., compressed temporal coupling), this strategy proved ineffective for maintaining spatial accuracy. Specifically, the spatial coupling error in the early occlusion condition did not significantly differ from the highest error observed in the both occlusions condition, and it was significantly greater than the lower error level seen in the late occlusion condition. This outcome highlights that, unlike temporal accuracy, spatial accuracy cannot be compensated for by mere temporal adjustments, emphasizing the essential role of predictive spatial planning based on early trajectory information (de Brouwer and Spering, 2022). Taken together, these results emphasize that while highly trained performers can flexibly adapt temporal aspects of visuomotor behavior, spatial precision remains strongly constrained by the availability of early information. In conclusion, this study demonstrates that successful visuomotor interception is achieved through the integrated coordination of both predictive and prospective control, and that the availability of early visual information appears to play an important role in maintaining spatial accuracy under heightened temporal constraints (Fiehler et al., 2019).

## 5 Conclusion

This study demonstrated that the visuomotor system dynamically adapts to temporal constraints and uncertainty in visual information through flexible strategic adjustments. All temporal variables consistently decreased as stimulus velocity increased, and distinct compensatory strategies were observed under conditions of partial visual occlusion. In the early occlusion condition, participants employed an early initiation strategy to shorten reaction time, whereas in the late occlusion condition, a completion timing adjustment strategy reduced movement time. Despite these differing approaches, both conditions achieved comparable levels of temporal coupling efficiency, suggesting that the visuomotor system can flexibly reallocate temporal resources to mitigate information loss. However, these timing adjustments did not significantly affect hand timing errors, indicating that manual temporal control is governed by an internal timing model that operates independently of immediate visual availability (Brenner and Smeets, 2015).

Despite the success of temporal compensation strategies, spatial accuracy measures—such as gaze error, radial error, and spatial coupling—were highly sensitive to the absence of early predictive information. Although the early occlusion condition exhibited prolonged fixation durations and compressed temporal coupling as compensatory attempts, these efforts were insufficient to offset spatial coupling errors. Moreover, both occlusions condition produced the greatest spatial disruption, suggesting that the simultaneous loss of predictive and online control fundamentally impaired spatial integration (Márquez et al., 2024; Sun and Jang, 2025). These findings indicate that spatial accuracy cannot be maintained through timing adjustment alone and instead depends critically on robust predictive spatial planning based on early trajectory information.

In summary, successful visuomotor interception requires the integrated coordination of predictive and prospective control, with early visual information being decisive for preserving spatial accuracy under temporal constraints. From a practical perspective, these findings suggest that sports training programs should prioritize the early acquisition of trajectory cues to enhance spatial precision, rather than relying solely on speed-based temporal adjustments. Furthermore, the observed compensatory strategies and their breakdown points could serve as a valuable basis for developing skill-assessment protocols that evaluate an athlete’s ability to maintain spatiotemporal synchronization under varying informational constraints.

Regarding the technical constraints of this study, several limitations should be acknowledged. The use of a two-dimensional touchscreen setup and the specific sampling rate of the eye-tracking system may not fully capture the high-velocity dynamics and three-dimensional complexity typical of real-world interception. Furthermore, the use of a chinrest to ensure high-precision eye-tracking restricted natural head movements, which are essential for gaze-stabilization in field-based sports (Mann et al., 2013). Additionally, although all participants were skilled collegiate baseball players, their familiarity with the medium velocity condition (0.67 m/s) may have induced partially automated responses, potentially reducing sensitivity to task-related manipulations. Lastly, while the number of trials for each of the 12 velocity-occlusion combinations (*n* = 10 per cell) was chosen to minimize participant fatigue and preserve performance quality, this may limit the ability to fully characterize the non-normal distribution of temporal variables such as reaction time. Future research involving a larger number of trials per cell or utilizing trial-by-trial distribution modeling would provide a more nuanced understanding of the temporal variability in skilled interceptive actions.

Future studies should address these limitations by employing virtual reality (VR) environments to replicate this task in a more realistic three-dimensional setting, thereby enhancing ecological validity and allowing for the examination of unconstrained eye-head coordination. Second, the inclusion of skill level (e.g., expert vs. novice) as an independent factor could clarify how automatic and cognitively controlled strategies manifest differently under information constraints. Finally, integrating neuroscientific approaches such as EEG or fMRI would allow for the identification of neural regions involved in the integration of predictive and corrective control mechanisms, deepening our understanding of the neural basis of visuomotor integration.

## Data Availability

The original contributions presented in the study are included in the article/supplementary material, further inquiries can be directed to the corresponding author.
